# Global excellence in rheumatology: Africa–The contribution of African women rheumatologists

**DOI:** 10.3389/fmed.2022.1032122

**Published:** 2022-11-25

**Authors:** Ihsane Hmamouchi, Adewale Adebajo

**Affiliations:** ^1^Rheumatology Health Sciences College, International University of Rabat (UIR), Rabat, Morocco; ^2^Laboratory of Biostatistical, Clinical and Epidemiological Research (LBRCE), Faculty of Medicine and Pharmacy, Rabat, Morocco; ^3^Faculty of Medicine, Dentistry and Health University of Sheffield, Sheffield, United Kingdom

**Keywords:** rheumatology, Africa, women leader, gender, research

## Abstract

Over the last few decades, the development of Rheumatology on the African continent has made great strides. Alongside an increasing recognition of the prevalence and burden of disease from these conditions, has been a determination to raise awareness of these disorders in Africa together with an appreciation of the associated disease impact on individuals, families and society as a whole. Great improvements have also been made across the continent with regards to the education of medical students, trainee doctors and allied health professionals of these conditions. Furthermore, high quality rheumatological research is now emanating from Africa. Taken together, these actions mean that Africa is making a significant contribution to global excellence in Rheumatology. Although this achievement has been made possible by visionary, hardworking and inspirational men and women, we believe that it is vital to bring to light the extraordinary efforts of African female rheumatologists in this endeavor. Their contribution is all the more remarkable because of the barriers that women still face in medicine in general and in academia in particular. We showcase here, four women of distinction who in their own unique way, have exemplified the contribution of African female rheumatologists to global excellence in Africa. We pay tribute to them and to their ongoing efforts to act as beacons of excellence in rheumatology in Africa to men and especially to other women.

## Introduction

Over the last few decades, the development of Rheumatology on the African continent has made great and impressive strides (1, 2). Alongside an increasing recognition of the prevalence and burden of disease from these conditions, has been a determination to raise awareness of these disorders in Africa together with an appreciation of the associated disease impact on individuals, families, and society as a whole. Great improvements have also been made across the continent with regard to the education of medical students, trainee doctors, and allied health professionals about these conditions. Furthermore, high-quality rheumatological research is now emanating from Africa (2).

Taken together, these actions mean that Africa is making a significant contribution to global excellence in Rheumatology. Although this achievement has been made possible by visionary, hardworking, and inspirational men and women, we believe that it is vital to bring to light the extraordinary efforts of African women rheumatologists in this endeavor. Their contribution is all the more remarkable because of the barriers that women still face in medicine in general and in academia in particular (3–5).

Indeed, in our recent survey of 2970 African Rheumatologists (adult and pediatric Rheumatologists), 1772 (60%) of them were women, 62% of whom were from North Africa. All countries except Uganda and Ethiopia had at least one female rheumatologist. Women were more often engaged in academics (71%) and public practice (65%) and were less frequently involved in the private sector (27%).

Of the 44 countries participating in the survey, 18 (41%) have a national society for Rheumatology, of which 5 (28%) are under the presidency of a woman (5).

We showcase here, four women of distinction who in their own unique way, have exemplified the contribution of African women rheumatologists to global excellence in Africa. We pay tribute to these women and to their ongoing efforts as beacons of excellence in rheumatology in Africa.

## Four examples of pillars in African rheumatology

### Professor Najia Hajjaj-Hassouni (Morocco)

Professor Najia Hajjaj-Hassouni, who hails from Morocco, is the first Professor of Rheumatology in Morrocco and created the first University department of Rheumatology in the country. She is also the fourth President of the African League of Associations for Rheumatology (AFLAR) and the first woman President of AFLAR (2007–2011), having previously served as the AFLAR Secretary General (1989–1995).

Professor Hajjaj-Hassouni graduated with honors from the Mohammed Vth University–Faculty of Medicine of Rabat and went on to choose to specialize in Rheumatology as she felt that Rheumatology was a real link between Internal Medicine and Radiology, combining both the richness as well as diversity of Internal Medicine with the analytical and interpretative abilities of Radiology. Professor Hajjaj-Hassouni trained in both Morocco and France and has held numerous senior academic positions including Director of the National Diploma for Specialty in Rheumatology (1998–2016) which enabled her to further the development of medical education and especially Rheumatology education. She has also used her appointments as Director of the Innovation Center and subsequently as Vice-President for Strategic Research and Development at Mohammed VI University of Health Sciences, Cassablanca, to transform Rheumatology research within Morocco and across North Africa. Professor Hajjaj-Hassouni has continued this important work in her position as the first Dean of a Faculty of Medicine and Pharmacy in Morocco.

Professor Hajjaj-Hassouni helped to found the Moroccan Society of Rheumatology in 1980 and has held the positions of Secretary General (1983–1986) and President (1988–1992; 1994–2003). As a result, she has organized the first 14 Congresses of the Society as well as an AFLAR Congress of Rheumatology. She was awarded Honorary Life Presidency of the society in January 2004. Other notable achievements include the creation of the first Moroccan Diploma for Ultrasound in Rheumatology and she established the first Moroccan Journal of Rheumatology (Revue Marocaine de Rhumatologie) in addition to being awarded the Gold Medal of the French Academy of Medicine (2010) and the Medal of National Merit of the Order Commander by His Majesty King Mohammed VI (2010). Professor Hajjaj-Hassouni was invited to speak at the 2021 American College of Rheumatology Congress together with three other women Rheumatologists, who were all identified as global women leaders in Rheumatology. Professor Hajjaj-Hassouni has published in numerous journals, reflecting her considerable research output which includes major international clinical studies (6–8).

Professor Hajjaj-Hassouni has encouraged women to pursue careers in medicine and in Rheumatology in particular. She believes that family is very important, but that it is possible for women to achieve a healthy balance between their professional and family life. Professor Hajjaj-Hassouni recognizes that Rheumatology remains a fragile specialty globally due to reductions in Rheumatology hospital beds and as a result of dominating pressure from other medical specialties. She is hopeful that women Rheumatologists will rise to this challenge and she is also encouraged by the fact that in many parts of the world, including Morocco, women are now the majority gender in annual graduands from medical schools.

### Professor Aicha Ladjouze (Algeria)

Professor Ladjouze hails from Algeria and is the second and the only other woman President of AFLAR. Professor Ladjouze graduated as a medical doctor and also trained in Rheumatology in Algeria. She chose to train in Rheumatology as a result of her contact with the first Maghrebian Rheumatologist. Professor Ladjouze has held various senior positions in Rheumatology, including Head of Department, Coordinator of the Clinical Experts in Rheumatology, President of the Regional and National Educational Committee of Rheumatology, and President of the Algerian League of Rheumatology.

Professor Ladjouze is an outstanding clinician, teacher, and researcher. Her research has led to several important publications (9–11). Professor Ladjouze has trained dozens of Rheumatologists, from all regions of Algeria and she has overseen over 10 Doctorate theses. Some of her trainees are now Heads of Rheumatology Departments. She has also facilitated the training of over 100 rheumatologists in musculoskeletal ultrasound. Professor Ladjouze has organized at least 18 National Congresses of Rheumatology as well as an international Mediterranean Rheumatology Conference and an international AFLAR Congress of Rheumatology.

Professor Ladjouze encourages women rheumatologists to be determined to prove that they are every inch the equal of their male counterparts, but also to be flexible, without compromising their dream. She believes that women Rheumatologists must avail themselves of digital technology to ensure their continuous professional development.

### Doctor Dzifa Dey (Ghana)

Doctor Dzifa Dey from Ghana, is the current Secretary General and Acting President Elect of AFLAR. Although she started off as a Nephrology Fellow, she found that her real passion was to adequately treat patients with Systemic Lupus Erythematosus, so as to stop these patients from developing Lupus Nephritis and eventually prevent End Stage Renal Disease. She became increasingly determined to improve the quality of care for patients with autoimmune rheumatic disorders in Ghana. Unfortunately, there were no existing Rheumatologists in Ghana. However, she was able to obtain an academic placement in Rheumatology at the prestigious University College Hospital in London. Dr. Dey subsequently returned to Ghana as the country's first Rheumatologist. She set up Rheumatology Clinics at Korlebu Teaching Hospital and a Rheumatology Department at the University of Ghana Medical School, where she is Head of Department. Dr. Day is also the Founder and Director of The Rheumatology Initiative, which has promoted vital autoimmune disease advocacy in Ghana and the West African sub-region. In addition, she is an Examiner for the West African College of Physicians subspecialty of Rheumatology and a volunteer for the LMS Task Force of the American College of Rheumatology. She has developed a Rheumatology Training program in Ghana and across the West African Sub-region.

Doctor Dey was the winner of the first ever distinguished international Rheumatology health professional award of the American College of Rheumatology. She has published widely in many peer-reviewed journals on a range of Rheumatology related topics, health disparities (10), and particularly systemic lupus erythematosus (12–14).

Doctor Dey believes that women have strong innate personal skills including attention to detail, fairness, and empathy which are all much-needed skills in medicine in general and Rheumatology in particular. Dr. Dey is confident that by their very nature, women Rheumatologists will have a tendency to nurture patients with musculoskeletal disorders and to invest in their care outcomes. Dr. Dey advises that women Rheumatologists should identify their champions and mentors early, as these will encourage and drive them forward to success. In turn, these women Rheumatologists will then be able to go on and provide mentoring and support to the next generation of women Rheumatologists. Dr. Dey wants more women Rheumatologists to step up and share their successes with both the scientific community and the world more broadly, so as to inspire other women.

### Professor Angela Migowa (Kenya)

Professor Angela Migowa is the first woman Pediatric Rheumatologist in Africa and she is a Professor of Pediatric Rheumatology in Kenya. She is also the Founder President of PAFLAR and Member of Association of Women in Rheumatology. She has published extensively in the field of pediatric rheumatology (15–17). She chose rheumatology because she was inspired by the presenters and presentations at an AFLAR Conference held in Kenya. She encountered many patients during her residency who were suffering from pediatric rheumatology diseases at McGill University Health Center, Quebec Canada. Prof Migowa felt motivated to help these children. Prof Migowa top achievements included the establishment of PAFLAR, PAFLAR Congresses, PAFLAR Monthly webinar series, and Establishment of Kenya Pediatric Rheumatology Registry (KAPRI Registry).

Regarding the role of women rheumatologists in contributing to excellence in rheumatology in Africa, Professor Migowa encourages women to understand and know themselves and to remain faithful and committed to a larger vision despite the various challenges. Professor Migowa believes that good things take time and that women rheumatologists should be patient with themselves and allow themselves to be a “work in progress,” so as to become the best version of themselves.

## Conclusion

In this article, we have paid tribute to the significant contribution of women Rheumatologists to global excellence in Rheumatology in and from Africa. African women Rheumatologists have clearly made huge impacts on research and education in Rheumatology. In addition, they have been instrumental in the development of the specialty of Rheumatology in Africa. Ziade and colleagues have recently emphasized the importance of gender issues in Rheumatology (18). The inadequate recognition of the role of women Rheumatologists has also recently been raised (19–21).

We believe that this article will assist with the global efforts to redress this unfairness. Furthermore, we believe and hope that both men and women from Africa, will continue to advance excellence in Rheumatology not only in Africa but also, across the world.

## Data availability statement

The original contributions presented in the study are included in the article/Supplementary material, further inquiries can be directed to the corresponding author.

## Ethics statement

Ethical review and approval was not required for the study on human participants in accordance with the local legislation and institutional requirements. Written informed consent from the (patients/ participants OR patients/participants legal guardian/next of kin) was not required to participate in this study in accordance with the national legislation and the institutional requirements. The four individuals identified here were invited to respond through personal email invitations and it was made clear that they would be identified. All four individuals accepted and chose to respond to the questionnaire.

## Author contributions

Both authors listed have made a substantial, direct, and intellectual contribution to the work and approved it for publication.

## Funding

We acknowledge open access funding from the University of Sheffield Institutional Open Access Fund. For the purpose of open access, the author has applied a Creative Commons Attribution (CC BY) license to any author accepted manuscript version arising.

## Conflict of interest

The authors declare that the research was conducted in the absence of any commercial or financial relationships that could be construed as a potential conflict of interest.

## Publisher's note

All claims expressed in this article are solely those of the authors and do not necessarily represent those of their affiliated organizations, or those of the publisher, the editors and the reviewers. Any product that may be evaluated in this article, or claim that may be made by its manufacturer, is not guaranteed or endorsed by the publisher.
