# Supplementary material for: Global excellence in rheumatology: Africa–The contribution of African women rheumatologists
Source: Front Med (Lausanne). 2022 Nov 25;9:1032122. doi: 10.3389/fmed.2022.1032122 (PMC9732090; doi:10.3389/fmed.2022.1032122)
Supplement: Supplementary file 1 [file Presentation_1.pdf]

Questionnaire

1) What made you choose Rheumatology as a specialty?

2) What training did you have in Rheumatology?

3) What positions have you held in a senior post in a pan-African Rheumatology organisation?

4) List your top three publications in Rheumatology.

5) List your top five achievements in Rheumatology

6) What additional comments do you have regarding the role of female Rheumatologists in contributing to Excellence in Rheumatology in Africa?

7) What advice do you have for young female doctors who are just starting a career in Rheumatology and who see you as a role model?
